# Radioimmunoluminography: a tool for relating tissue antigen concentration to clinical outcome

**DOI:** 10.1038/sj.bjc.6690443

**Published:** 1999-05

**Authors:** G Boxer, S Stuart-Smith, A Flynn, A Green, R Begent

**Affiliations:** Cancer Research Campaign Targeting and Imaging Group, Department of Oncology, Royal Free and University College Medical School, Rowland Hill Street, London, NW3 2PF, UK

**Keywords:** quantification, immunohistochemistry, radioluminography, phosphor image, colorectal cancer, targeting

## Abstract

Proteins control cell function and measurement of their concentration is crucial for understanding their roles in health and disease. However, current methods for their detection in tissue sections are not quantitative. Radioimmunoluminography (RILG) is a system for direct measurement of quantity and distribution of protein in histological sections. Histological carcinomas were reacted with antigen-saturating concentrations of ^125^I-antibody to carcinoembryonic antigen (CEA). Antibody distribution and concentration was mapped by phosphor imaging. Radioactivity in each pixel of the digital image was proportional to antigen concentration, calculated from a standard line generated from a nitrocellulose CEA dot-blot assay. RILG gave a linear correlation with standards of known CEA concentration (*r* = 0.999). Six tumour xenografts with differing CEA concentrations by radioimmunoassay (RIA) were studied by RILG and immunohistochemistry (IHC). RILG gave a linear correlation with CEA by RIA (*r* = 0.994) but IHC failed to do so (*r* = 0.42). CEA levels measured by RILG, in cryostat (*n* = 15) and paraffin (*n* = 19) sections from colorectal cancer patients showed a range of CEA concentration (38.9–594 ng g^−1^ and 22.5–212.5 ng g^−1^ respectively). Tumour CEA concentration by RILG was significantly correlated with dose of antibody (% injected radioactivity kg^−1^) localized in tumour (*P* = 0.04 and *P* < 0.02 respectively), in patients receiving radioimmunoguided surgery. Clinical relevance of RILG is illustrated by identifying patients with high tumour CEA, most likely to benefit from antibody targeted therapy. Knowledge of the pathophysiology of many diseases may be enhanced by quantitative estimation of antigen concentration related to tissue morphology. © 1999 Cancer Research Campaign


*British Journal of Cancer* (1999) **80**(5/6), 922–926
© 1999 Cancer Research Campaign
Article no. bjoc.1998.0443

### Radioimmunoluminography: a tool for relating tissue antigen concentration to clinical outcome

**G Boxer, S Stuart-Smith, A Flynn, A Green and R Begent**

Cancer Research Campaign Targeting and Imaging Group, Department of Oncology, Royal Free and University College Medical School, University College London, Rowland Hill Street, London NW3 2PF, UK

**Summary** Proteins control cell function and measurement of their concentration is crucial for understanding their roles in health and disease. However, current methods for their detection in tissue sections are not quantitative. Radioimmunoluminography (RILG) is a system for direct measurement of quantity and distribution of protein in histological sections. Histological carcinomas were reacted with antigen-saturating concentrations of $^{125}$I-antibody to carcinoembryonic antigen (CEA). Antibody distribution and concentration was mapped by phosphor imaging. Radioactivity in each pixel of the digital image was proportional to antigen concentration, calculated from a standard line generated from a nitrocellulose CEA dot-blot assay. RILG gave a linear correlation with standards of known CEA concentration ($r = 0.999$). Six tumour xenografts with differing CEA concentrations by radioimmunoassay (RIA) were studied by RILG and immunohistochemistry (IHC). RILG gave a linear correlation with CEA by RIA ($r = 0.994$) but IHC failed to do so ($r = 0.42$). CEA levels measured by RILG, in cryostat ($n = 15$) and paraffin ($n = 19$) sections from colorectal cancer patients showed a range of CEA concentration (38.9–594 ng $g^{-1}$ and 22.5–212.5 ng $g^{-1}$ respectively). Tumour CEA concentration by RILG was significantly correlated with dose of antibody (% injected radioactivity $kg^{-1}$) localized in tumour ($P = 0.04$ and $P < 0.02$ respectively), in patients receiving radioimmunoguided surgery. Clinical relevance of RILG is illustrated by identifying patients with high tumour CEA, most likely to benefit from antibody targeted therapy. Knowledge of the pathophysiology of many diseases may be enhanced by quantitative estimation of antigen concentration related to tissue morphology.

**Keywords:** quantification; immunohistochemistry; radioluminography; phosphor image; colorectal cancer; targeting

Many diseases are characterized by altered expression of proteins or peptide antigens which define diagnosis or disease behaviour. The biological significance of an antigen is generally dependent on its concentration and there is a need for a means of measuring antigen concentration in tissue sections. Visualization of tissue morphology is important because antigen expression is commonly related to a particular cell population within a mixed tissue. Immunohistochemistry (IHC) retains tissue morphology but does not reliably quantitate antigen while assays of extracts of homogenized tissue involve destruction of morphology.

The principal aim of this work was, first, to establish and validate a method for quantifying protein concentration in histological sections of colorectal cancer and then, second, to apply this technique to a clinically relevant situation.

Radioimmunoluminography (RILG), a generally applicable method for measurement of antigen concentration in tissue sections, is based on quantification of radiolabelled antibody binding to tissue sections using a phosphor plate imager. The amount of antibody bound in a given area of the section will be proportional to the amount of antigen present if saturating concentrations of antibody are used. The distribution of radioactivity in a section is mapped by a phosphor plate imager which quantitates antigen-bound antibody pixel by pixel in the digital image. The high sensitivity and linear response over five orders of magnitude of this instrument (Chung et al, 1994; Okuyama et al, 1994) make it practical to quantitate the very small amounts of radioactivity present. The pixel size of available phosphor imagers (25–90 μm) gives sufficient resolution for correlation with light microscope images. Here we show that the concentration of carcinoembryonic antigen (CEA) in histological sections can be quantitated using a calibration line constructed from RILG binding to known amounts of CEA. The accuracy of RILG has been established by assessing CEA levels in sections of human colon carcinoma xenografts.

In antibody-directed targeted therapy of cancer the localization of anti-tumour antibodies in tumours varies greatly between individual patients and as yet the factors responsible for this variation have not been critically defined (Boxer et al, 1992). Whilst there have been a number of papers implicating a variety of putative cellular and physiological factors (Jain, 1989; Poznansky and Juliano, 1984; Shockley et al, 1991) there has been surprisingly little attention paid to the importance of tumour antigen concentration. The ability of RILG to directly measure CEA concentration in biopsies is shown to be clinically useful and can enable us to predict the likely efficiency of antibody-targeted therapy of colorectal carcinoma.

### MATERIALS AND METHODS

### Tissue preparation

Human adenocarcinoma xenografts (Pedley et al, 1996) with different levels of CEA expression, SW116, LS174T(1), LS174T(2), HT29/219 (colon), OVCA (ovarian), PAPA (breast) and a CEA-negative, rat mammary (RM) tumour, were used to

*Received 13 July 1998*
*Accepted 17 November 1998*

*Correspondence to:* G Boxer

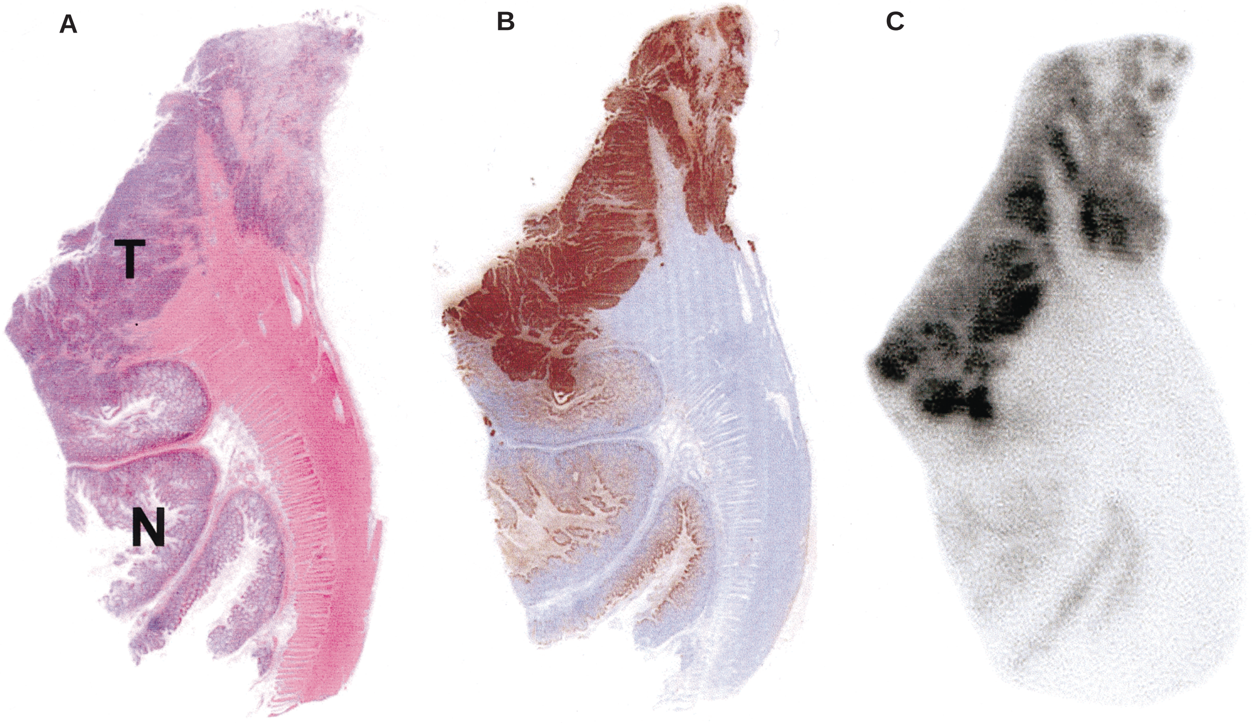

**Figure 1** Images of formalin-fixed paraffin sections of a tumour (T), a human colorectal adenocarcinoma and adjacent non-neoplastic mucosa (N), (**A**) stained by haematoxylin and eosin, (**B**), (**C**) reacted for CEA by IHC and RILG respectively. CEA is shown by a brown reaction product with IHC and by scales of grey with RILG. Unlike IHC, the RILG data is digital and also gives a qualitative assessment of CEA distribution establish the technique. Fresh samples of tumour xenograft were snap-frozen in iso-pentane (cooled in liquid nitrogen) and 6-µm cryostat sections were prepared. For the clinical study sections were cut from colorectal carcinomas resected from patients receiving radioimmunoguided surgery (Blair et al, 1990). Tumour from 15 patients was assayed for the cryostat study (6-µm sections) and from 19 patients for the paraffin study (3-µm sections). All sections were mounted on slides coated with 2% 3-aminopropyltriethoxysilane (Sigma-Aldrich Ltd, U.K.) in acetone.

### Antibodies

A5B7 anti-CEA (Harwood et al, 1986) and A161 (Hitchens et al, 1989) anti-alphafetoprotein antibodies were labelled (Fraker and Speck, 1978) with $^{125}$I (21.5–120.5 MBq mg$^{-1}$ and 104 MBq mg$^{-1}$ respectively) passed through a 0.22 µm filter (Gellman Ltd, Northampton, UK). Percentage incorporation of $^{125}$I into antibody was calculated, from thin layer chromatographs (TLC) on 0.25-mm silica gel (Macherey-Nagel, Polytron sil G/UV 254), using phosphor image plate technology (see later).

### Radioimmunoassay

A total of 0.2–1.0 g of each tumour xenograft was homogenized in 5 ml of buffer with protease inhibitors; 4-(2-aminoethyl)-benzene-sulphonyl fluoride hydrochloride (ICN, UK); pepstatin A and leupeptin (Sigma, Poole, UK) and incubated for 2 h at 37°C in phosphatidyl-inositol-specific phospholipase C (Sigma, UK) (2.95 units g$^{-1}$ wet weight). After mixing, by vortexing, tubes were incubated for 2 h at 37°C in a shaking incubator (New Brunswick Scientific Ltd, UK) at 200 oscillations min$^{-1}$. The preparation was centrifuged for 30 min at 14 300 ***g*** at 4°C in a high speed centrifuge (Beckman JA20.1; Beckman Ltd, UK) to remove all the cell debris. Supernatant was removed and stored at 4°C and the pellet was resuspended in 5 ml of extraction buffer, mixed thoroughly by vortexing and centrifuged for a further 30 min at high speed (14 300 ***g***). The supernatant was removed and stored at − 70°C. Samples were assayed for CEA using the solid phase two-site (ELSA-2) radioimmunoassay (RIA) kit (CIS International UK Ltd) at neat, 1/10, 1/100 and 1/1000 dilutions. Each tube was counted using a gamma counter (WIZARD; Pharmacia, UK). Values for CEA for each tumour were expressed in ng g$^{-1}$ of wet weight of tissue calculated with reference to a CEA standard line produced using CEA standards from the commercial RIA kit.

### Nitrocellulose dot blot assay

A total of 0.45-µm nitrocellulose Trans-blot membrane (Biorad UK Ltd) was dotted with 10-µl aliquots of 0.05–5 µg g$^{-1}$ CEA and allowed to air-dry for 30 min. The nitrocellulose was cut into squares containing individual dots of antigen and assays performed using 12-well plastic plates (Costar Ltd, UK). After blocking with 5% bovine serum albumin in Tris–HCl buffered saline pH 7.6 containing 1% normal horse serum for 1 h, 650 µl of $^{125}$I-labelled A5B7 anti-CEA antibody (75 µg g$^{-1}$) was added to each well containing a nitrocellulose dot and incubated for 75 min at room temperature (RT). After washing in five changes of phosphate-buffered saline (PBS)–Tween (0.05%) nitrocellulose

*© Cancer Research Campaign 1999*

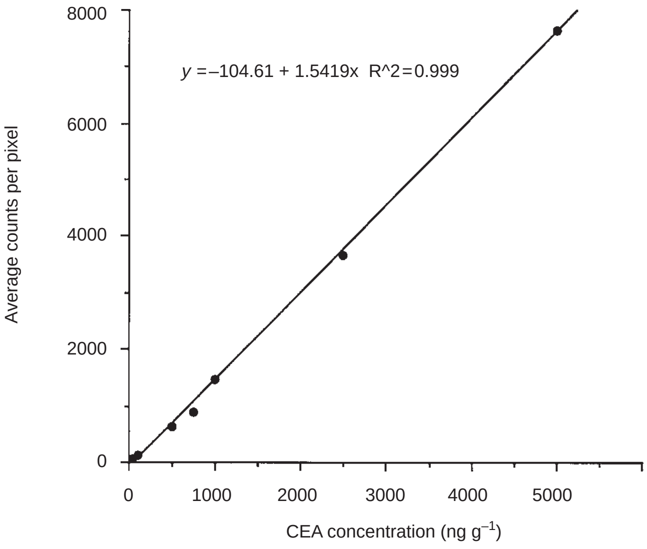

**Figure 2** Binding of $^{125}$I anti-CEA antibody by RILG to known concentrations of CEA, in a nitrocellulose dot blot assay

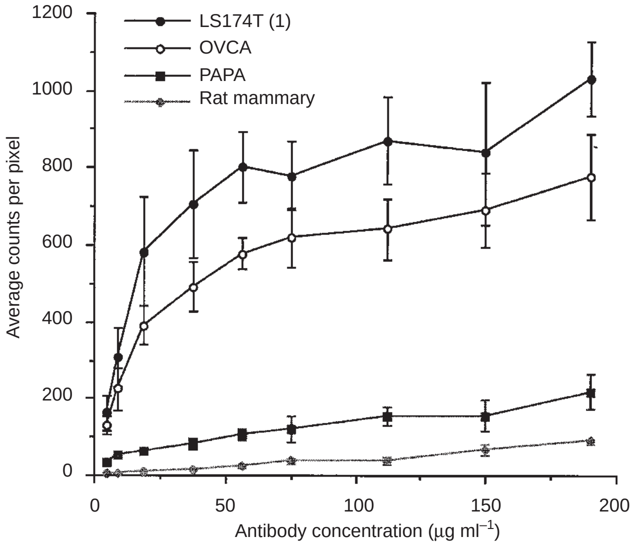

**Figure 3** Binding of $^{125}$I anti-CEA antibody (concentration range 4.7–190 µg ml$^{-1}$) by RILG to three CEA-expressing xenografts (LS174T(1), OVCA and PAPA) and non CEA-expressing rat mammary carcinoma squares were air-dried and exposed overnight to a phosphor storage plate before scanning (400-A; Molecular Dynamics, UK).

### Immunohistochemistry

Immunohistochemistry (IHC) was carried out as described previously (Southall et al, 1990) using an avidin–biotin–peroxidase technique. Briefly, 5-µm cryostat sections of xenograft tumours were air-dried, fixed in acetone and reacted with biotinylated A5B7 anti-CEA antibody (Marshall et al, 1995) at 20 µg ml$^{-1}$ and visualized using avidin–biotin–peroxidase complexes (Vector Laboratories, UK). Under bright-field microscopy immunohistochemically reacted sections were analysed and measurement of CEA content was estimated by screening 20 randomly selected high power fields (magnification × 200) per xenograft tumour and calculating the mean % CEA-reactive cells. For both the cryostat and paraffin sections from cases of human tumour, following the RILG assay, CEA antigen distribution was demonstrated, by incubation with biotinylated horse anti-mouse immunoglobulins and avidin–biotin–peroxidase complexes sequentially. Endogenous peroxidase was blocked using a solution of 0.3% hydrogen peroxide (Merck Ltd, UK) in methanol (Merck Ltd, UK).

### RILG

Cryostat sections were fixed in 0.25% glutaraldehyde (40 min). Cryostat and dewaxed paraffin sections were blocked in 5% bovine serum albumin (BSA) in Tris–HCl buffered saline, pH 7.6 containing 1% normal horse serum. Binding of $^{125}$I-labelled A5B7 antibody (0.01–120 µg ml$^{-1}$ and subsequently 4.7–190 µg ml$^{-1}$) to cryostat sections of xenografted tumours (CEA concentration range 0.5–935 ng g$^{-1}$ as determined by RIA) was measured using phosphor image analysis. RM tumour and A161 anti-AFP antibody were included as negative controls. Experiments were performed in triplicate on three separate days to determine the inter-assay variation. Antibody binding to sections of the xenografts plateaued at approximately 60–75 µg ml$^{-1}$ (see Results and Figure 3) and 75 µg ml$^{-1}$ was chosen as saturating concentration of antibody for subsequent work. Slides were incubated with $^{125}$I antibody for 1 h at RT, washed in five changes of Tris–HCl buffer and air-dried. Slides were exposed to phosphor storage plates for 1 h before scanning.

### Quantitation of RILG

Binding of radiolabelled antibody to tumours was measured by demarcating regions of interest. Regions were drawn using automated image analysis techniques (ImageQuant NT software for Windows). Average counts per pixel were taken for the region and a mean from triplicate sections used to generate antibody binding curves. RILG values for xenografts and for all regions identified as tumour from patients' studies were recorded as average counts per pixel and converted to ng g$^{-1}$ of CEA using the relevant part of the CEA standard calibration line (0–1000 ng g$^{-1}$). Statistical analyses were performed using a non-parametric test (Spearman rank correlation test).

### RESULTS

### RILG images

Anti-CEA antibody binding to CEA by RILG in a section of colon carcinoma is shown in Figure 1C. The number of counts in each pixel of the image is directly related to the amount of $^{125}$I antibody bound to CEA. IHC (Figure 1B) and haematoxylin and eosin (Figure 1A) stained sections of the same tumour are shown for comparison. RILG has the advantage of illustrating the heterogeneity of CEA expression within tumours, something which is not demonstrable by traditional IHC methods. The range of counts per pixel measured within a tumour region is evidence of heterogeneity of protein expression within the tumour mass. This is also evident on visual inspection of RILG images (Figure 1C).

 © Cancer Research Campaign 1999

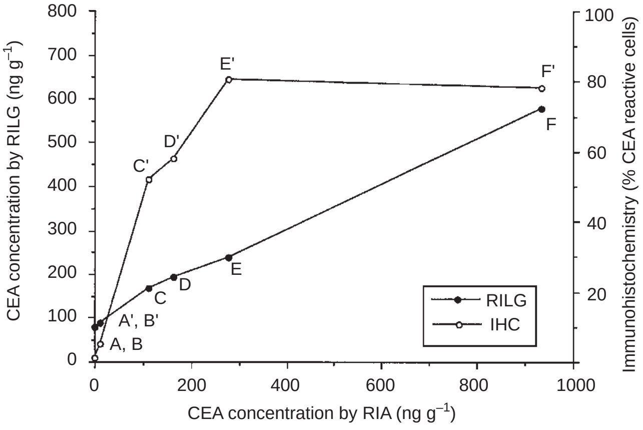

**Figure 4** Comparison of RILG and IHC in relation to CEA measured by radioimmunoassay of tumour homogenates in xenografts with different concentrations of CEA. Xenografts were: A,A′ – SW116; B,B′ – PAPA; C,C′ – OVCA; D,D′ – LS174T; E,E′ – LS174T; F,F′ – HT29/219

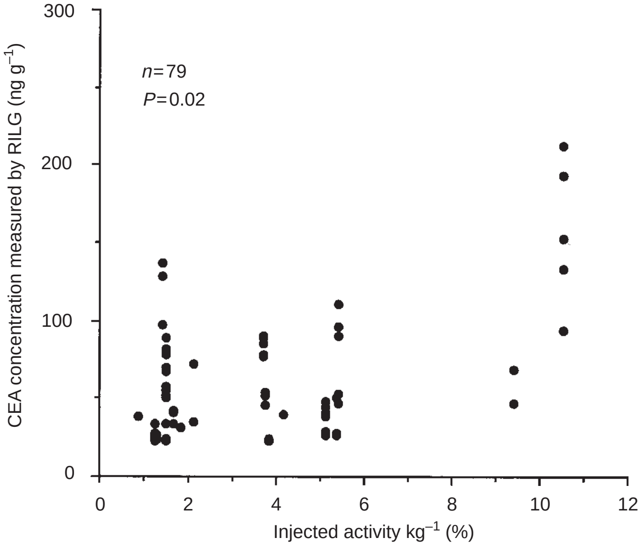

**Figure 5** Relationship between tumour CEA concentration measured by RILG (paraffin sections) and % of injected activity localizing in tumour in 19 patients ($n$ = 79 regions, $P = < 0.02$)

### Quantitative analysis

The relationship of antibody binding to antigen was shown by RILG to give a linear correlation ($r$ = 0.999) between $^{125}$I anti-CEA and concentrations of CEA ranging from 0.05–5 μg g$^{-1}$ in the nitrocellulose dot blots (Figure 2). Figure 3 shows the binding curves for three of the xenograft tumours (LS174T (1), OVCA and PAPA) and the control rat mammary carcinoma with increasing concentration of $^{125}$I-antibody. After an initial rise in counts per pixel with concentration, a plateauing effect is observed at approximately 60–75 μg ml$^{-1}$. These data come from experiments repeated on three successive days and the standard deviation for each concentration is shown. Binding to non-CEAexpressing rat mammary tumour was consistently very low and always below that observed for even the lowest CEA-expressing tumours as demonstrated in Figure 3. Tumour CEA concentrations measured by RIA of tissue extracts from carcinoma xenografts were compared with assessments of tumour CEA by % positive cells using IHC and mean counts per pixel on RILG. Results shown in Figure 4 demonstrate that IHC and RILG were each able to identify the low CEA-expressing (SW116) and high CEA-expressing (HT29/219) tumours, but whilst RILG could accurately show the intermediate level of CEA production IHC was unable to discriminate between intermediate and high levels of expression. The linear relationship of antibody binding to antigen in xenografts is demonstrated by the regression line for the RILG data ($r$ = 0.994) compared to that for IHC ($r$ = 0.47). Control RILG assays incubating sections of xenografts with A161 anti-AFP antibody, and rat mammary tumour with A5B7 anti-CEA antibody, always gave results (data not shown) below that of the lowest CEA expressing tumour (SW116). The CEA concentration of each xenograft calculated by RILG (78–578 ng g$^{-1}$) was closely correlated ($P$ = 0.025) to that calculated by RIA (0.5–935 ng g$^{-1}$).

### Clinical application

CEA levels estimated by RILG of cryostat sections of colorectal carcinomas in 15 patients (30 regions) ranged from 38.9 to 594 ng g$^{-1}$ and for formalin-fixed paraffin study of 19 patients (79 regions) between 22.5 and 212.5 ng g$^{-1}$. The correlation of RILG with parameters relevant to clinical management was assessed by comparing these values with levels of tumour localization of intravenously administered anti-CEA antibody in patients with colorectal cancer who received radiolabelled antibody for radioimmunoguided surgery. For both the cryostat study (15 patients) and paraffin study (19 patients), a significant statistical correlation was demonstrated between the percentage of injected radioactivity localizing in tumour (0.5–10.6% and 0.9–10.6%) and for CEA content ($P$ = 0.04 and $P$ = 0.02 respectively). Figure 5 shows the data for the paraffin study.

### DISCUSSION

This paper establishes a technique that uniquely combines measurement of concentration of cellular products with preservation of tissue morphology, allowing the heterogeneity of protein expression to be examined quantitatively. Currently, quantitative ways to measure tissue proteins by methods such as RIA after extraction, are limited by their failure to preserve cellular structure. Conversely, cellular techniques that maintain tissue architecture, such as IHC for the demonstration of antigen, are not quantitative. In this study the tumour antigen CEA was used to establish RILG for human colorectal tissues. Positive and negative antibody and antigen controls confirmed the specificity of RILG. IHC was shown to be non-quantitative for CEA in that it was unable to distinguish high from intermediate levels of CEA reflecting the non-linearity of the colourimetric amplification system. By contrast, the phosphor imaging system employed in RILG gives highly efficient energy capture (detection threshold for $^{125}$I is 0.23 dpm mm$^{2}$ h$^{-1}$) and is linear over five orders of magnitude (Chung et al, 1994; Okuyama et al, 1994), so that direct detection of radiolabelled antibody or other ligand is achieved without an amplification step. Antigen concentration is then directly related to the amount of radiolabelled antibody bound when antigen is saturated. Sensitivity was easily sufficient for the range of CEA concentrations studied here (0.49–935 ng g$^{-1}$), but

© Cancer Research Campaign 1999

the full limits of sensitivity have not been explored and will probably vary from one antigen to another.

Having established RILG in experimental models we then tested its utility in a clinical setting. The range of RILG CEA concentrations from the patients' study in cryostat section was similar to the range for the xenografts. RILG CEA concentrations for the paraffin section study of patients were generally at a lower level than those for the cryostat study. The lower level antibody binding may be due to prolonged formalin fixation or to histological processing, or be related to the lesser thickness of section (3 μm vs 6 μm).

Phosphor imaging technology can also be used to quantitate fluorescence and it is reasonable to suppose that this could also be used for measuring tissue antigen concentrations. The pixel size of current phosphor imagers (25–90 μm) enables phosphor plate and light microscope images to be correlated. Groups of cells and tissues can be readily investigated, but the resolution is not sufficient for single-cell studies. These characteristics are particularly valuable when studying conditions with heterogeneous antigen expression, as seen in neoplastic and inflammatory pathology, and gives insights not available from antigen measurements on tissue homogenates. The value of this is illustrated by showing that CEA concentration in areas of viable tumour is one parameter which predicts the amount of intravenously administered antibody which localizes in tumour. IHC had previously failed to demonstrate this (Boxer et al, 1992). Tumour responses are reported in patients treated with $^{131}$I-antibody to CEA (Lane et al, 1994) and antibody-directed enzyme prodrug therapy – ADEPT (Bagshawe et al, 1995). RILG of tumour biopsies is likely to assist in selecting those patients likely to respond to these treatments. The variation within the data in Figure 5 supports the hypothesis that there are a number of other cellular and physiological parameters (Jain, 1989; Poznansky & Juliano, 1984; Shockley et al, 1991) affecting antibody localization. One such putative factor is the vascular supply of tumours and this could also be studied by RILG.

This methodology could be adapted for direct quantitation of a wide range of tissue antigens, cell surface receptors, mRNA and viral RNA/DNA in histological sections leading to improved diagnosis of disease, rational selection of patients for appropriate therapy and better predictions of prognosis. The next phase of the Human Genome Project will concentrate on functional analysis of genes by studying their expression as proteins. This should lead to a deeper understanding of normal cell function and pathological states. In this context, the ability of RILG to accurately measure the concentrations of proteins at the same time as retaining their positions in tissues will contribute to knowledge of how cells function.

### ACKNOWLEDGEMENTS

This work was supported by the Cancer Research Campaign and the Ronald Raven Chair in Clinical Oncology Trust. Radiolabelling of antibodies to CEA was performed by Dr Joanne Casey and Dr Diane Marshall. We thank Celltech Ltd, UK for supplying A5B7 antibody to CEA. We are grateful to Ms J Lewin for the generation of the digitized colour images. The authors wish to thank Dr Kerry Chester for her advice.

### REFERENCES

Bagshawe KD, Sharma SK, Springer CJ and Antoniw P (1995) Antibody directed enzyme prodrug therapy: a pilot scale clinical trial. *Tumour Targeting* **1**: 17–29

Blair SB, Theodorou NA, Begent RHJ, Dawson PM, Salmon M, Riggs S, Kelly AMB, Boxer GM, Southall PJ and Gregory PA (1990) Comparison of anti-fetal colonic microvillus and anti-CEA antibodies in peroperative radioimmunolocalisation of colorectal cancer. *Br J Cancer* **61**: 891–894

Boxer GM, Begent RHJ, Kelly AMB, Southall PJ, Blair SB, Theodorou NA, Dawson PM and Lederman JA (1992) Factors influencing variability of localisation of antibodies to CEA in patients with colorectal carcinoma – implications for radioimmunotherapy. *Br J Cancer* **65**: 825–831

Chung J-K, Jang J-J, Lee D-S, Lee M-C and Koh C-S (1994) Tumour concentration and distribution of carcinoembryonic antigen measured by in vitro quantitative autoradiography. *J Nucl Med* **34**: 1499–1505

Fraker PJ and Speck JC (1978) Protein and cell membrane iodinations with a sparingly soluble chloroamide, 1,3,4,6-tetrachloro-3a,6a-diphenylglycoluril. *Biochem Biophys Res Commun* **80**: 849–857

Harwood PJ, Britton DW, Southall PJ, Boxer GM, Rawlins G and Rogers GT (1986) Mapping epitopes on carcinoembryonic antigen. *Br J Cancer* **54**: 75–82

Hitchens RN, Begent RHJ, Green AJ, Searle F, Van Heyningen V and Bagshawe KD (1989) Clinical value of imaging using antibody to alphafetoprotein in germ cell tumours. *Nuklear Medizin* **28**: 29–33

Jain RJ (1989) Delivery of novel therapeutic agents in tumours: physiological barriers and stratagies. *J Natl Cancer Inst* **81**: 570–576

Lane DM, Eagle KF, Begent RHJ, Hope-Stone LD, Green AJ, Casey JL, Keep PA, Kelly AMB, Lederman JA, Glaser MG and Begent RHJ (1994) Radioimmunotherapy of metastatic colorectal tumours with I-131 labelled antibody to CEA: phase I/II study with comparative biodistribution of intact and F(ab′)$_2$ antibodies. *Br J Cancer* **70**: 521–525

Marshall D, Pedley RB, Melton RG, Boden JA, Boden R and Begent RHJ (1995) Galactosylated streptavidin for improved clearance of biotinylated intact and F(ab′)$_2$ fragments of an anti-tumour antibody. *Br J Cancer* **71**: 18–24

Okuyama M, Hatori Y and Shigematsu A (1994) Autoradioluminography, a novel quantitative method of TLC-autoradiography. *Biol Pharm Bull* **17**: 559–563

Pedley RB, Boden JA, Boden R, Boxer GM, Flynn AA, Keep PA and Begent RHJ (1996) Ablation of colorectal xenografts with combined radioimmunotherapy and tumour blood flow-modifying agents. *Cancer Res* **56**: 3293–3300

Poznansky MR and Juliano RL (1984) Biological approaches to the controlled delivery of drugs: a critical review. *Pharmacol Rev* **36**: 277–336

Shockley TR, Lin K, Nagy A, Tompkins RG, Dvorak HF and Yarmush ML (1991) Penetration of tumour tissue by antibodies and other immunoproteins. *Ann NY Acad Sci* **618**: 367–381

Southall PJ, Boxer GM, Bagshawe KD, Hole N, Bromley M and Stern P (1990) Immunohistologic distribution of 5T4 antigen in normal and malignant tissues. *Br J Cancer* **61**: 89–95

© Cancer Research Campaign 1999

## References

[PDF_00412] Blair S. D., Theodorou N. A., Begent R. H., Dawson P. M., Salmon M., Riggs S., Kelly A., Boxer G., Southall P., Gregory P. (1990). Comparison of anti-fetal colonic microvillus and anti-CEA antibodies in peroperative radioimmunolocalisation of colorectal cancer.. Br J Cancer.

[PDF_00416] Boxer G. M., Begent R. H., Kelly A. M., Southall P. J., Blair S. B., Theodorou N. A., Dawson P. M., Ledermann J. A. (1992). Factors influencing variability of localisation of antibodies to carcinoembryonic antigen (CEA) in patients with colorectal carcinoma--implications for radioimmunotherapy.. Br J Cancer.

[PDF_00420] Chung J. K., Jang J. J., Lee D. S., Lee M. C., Koh C. S. (1994). Tumor concentration and distribution of carcinoembryonic antigen measured by in vitro quantitative autoradiography.. J Nucl Med.

[PDF_00423] Fraker P. J., Speck J. C. (1978). Protein and cell membrane iodinations with a sparingly soluble chloroamide, 1,3,4,6-tetrachloro-3a,6a-diphrenylglycoluril.. Biochem Biophys Res Commun.

[PDF_00426] Harwood P. J., Britton D. W., Southall P. J., Boxer G. M., Rawlins G., Rogers G. T. (1986). Mapping epitope characteristics on carcinoembryonic antigen.. Br J Cancer.

[PDF_00428] Hitchins R. N., Begent R. H., Green A. J., Searle F., van Heyningen V., Bagshawe K. D. (1989). Clinical value of imaging using antibody to alpha fetoprotein in germ cell tumours.. Nuklearmedizin.

[PDF_00431] Jain R. K. (1989). Delivery of novel therapeutic agents in tumors: physiological barriers and strategies.. J Natl Cancer Inst.

[PDF_00433] Lane D. M., Eagle K. F., Begent R. H., Hope-Stone L. D., Green A. J., Casey J. L., Keep P. A., Kelly A. M., Ledermann J. A., Glaser M. G. (1994). Radioimmunotherapy of metastatic colorectal tumours with iodine-131-labelled antibody to carcinoembryonic antigen: phase I/II study with comparative biodistribution of intact and F(ab')2 antibodies.. Br J Cancer.

[PDF_00439] Marshall D., Pedley R. B., Melton R. G., Boden J. A., Boden R., Begent R. H. (1995). Galactosylated streptavidin for improved clearance of biotinylated intact and F(ab')2 fragments of an anti-tumour antibody.. Br J Cancer.

[PDF_00443] Okuyama M., Hatori Y., Shigematsu A. (1994). Autoradioluminography, a novel quantitative method of TLC-autoradiography.. Biol Pharm Bull.

[PDF_00445] Pedley R. B., Boden J. A., Boden R., Boxer G. M., Flynn A. A., Keep P. A., Begent R. H. (1996). Ablation of colorectal xenografts with combined radioimmunotherapy and tumor blood flow-modifying agents.. Cancer Res.

[PDF_00448] Poznansky M. J., Juliano R. L. (1984). Biological approaches to the controlled delivery of drugs: a critical review.. Pharmacol Rev.

[PDF_00450] Shockley T. R., Lin K., Nagy J. A., Tompkins R. G., Dvorak H. F., Yarmush M. L. (1991). Penetration of tumor tissue by antibodies and other immunoproteins.. Ann N Y Acad Sci.

[PDF_00453] Southall P. J., Boxer G. M., Bagshawe K. D., Hole N., Bromley M., Stern P. L. (1990). Immunohistological distribution of 5T4 antigen in normal and malignant tissues.. Br J Cancer.

